# Impact of autologous platelet-rich plasma used for treatment of oligozoospermia in dogs on the quality of semen and testicular blood flow

**DOI:** 10.1007/s11259-025-10734-8

**Published:** 2025-05-01

**Authors:** Alaa Mohamed, Mohamed Fathi, Ashraf A. Shamaa, K. H. El Shahat

**Affiliations:** 1 Theriogenology Department, Faculty of Veterinary Medicine, Cairo University, Giza, 12211 Egypt; 2 Surgery, Anesthesiology and Radiology Department, Faculty of Veterinary Medicine, Cairo University, Giza, 12211 Egypt

**Keywords:** Autologous PRP, Semen evaluation, Testosterone, Nitric oxide, Doppler indices

## Abstract

Platelet-rich plasma (PRP) is widely used in regenerative medicine, and the current study aimed to investigate the effects of autologous PRP on semen characteristics, testicular blood flow, and testosterone levels in the treatment of oligozoospermia in dogs. Ten stray male dogs diagnosed with oligozoospermia were included in the study. The dogs were randomly assigned to two groups: Group I, the control group (*n* = 5), which received no treatment, and Group II, the PRP group (*n* = 5), which received a single intra-testicular injection of 0.5 mL of autologous PRP into each testicle. Testicular hemodynamics, hormonal and biochemical analysis and semen parameters were assessed for both groups and the examination was extended to 8 weeks. The results demonstrated that dogs treated with PRP showed significantly higher values of End Diastolic Velocity (EDV) and Peak Systolic Velocity (PSV) (*P* < 0.001) at the 6^th^ to 8^th^ weeks post-injection, compared to baseline (day 0) values. Conversely, the values of Resistance Index (RI) and Pulsatility Index (PI) showed a significant decrease (*P* < 0.001) in group II from week 2 to week 8. Additionally, PRP treatment led to significant increases in sperm concentration, motility percentage, and the proportion of live and normal spermatozoa (*P* < 0.001), with maximum values observed at 28 and 60 days post-treatment, compared to day 0. Moreover, serum testosterone and nitric oxide (NO) levels were significantly higher (*P* < 0.001) in group II and remained elevated through the 8^th^ week following injection, compared to baseline values and group I. In conclusion, this study demonstrates that autologous PRP treatment effectively increases sperm concentration, motility, and normal spermatozoa, improves testicular blood flow, and elevates testosterone and NO levels. These findings suggest that PRP may be a promising therapeutic option for the treatment of oligozoospermia in dogs. Further studies are needed to confirm and expand upon these results.

## Introduction

In clinical practice, male dog infertility is becoming increasingly significant. The reasons for infertility in males are not well understood. The two categories of reasons are acquired and congenital infertility. While acquired infertility develops after an animal has been fertile, congenital abnormalities appear from birth (Gobello and Corrada 2004; Fontbonne 2011). Many conditions, including prostatic and testicular problems, hormonal imbalances, infectious diseases, certain drugs, and others, can result in acquired infertility (Gobello and Corrada 2004; Gradil et al. 2006; Memon 2007; Fontbonne 2011; Schäfer-Somi 2015). The reason behind male canine infertility is frequently still unknown (Johnston et al. 2001a; Fontbonne 2011). Several problems, such as low-quality semen, might cause it (Mickelsen et al. 1993; Feldman and Nelson 1996; Fontbonne 2011; Domosławska et al. 2013).

During puberty, the hypothalamic-pituitary-gonadal (HPG) axis plays a central role in the regulation of testosterone levels and gonadal function. The hypothalamus secretes gonadotropin-releasing hormone (GnRH), which is secreted in a pulsatile way and transported through the hypothalamohypophyseal portal system to the anterior pituitary gland (Reece et al. 2015). In response, the pituitary releases luteinizing hormone (LH) and follicle-stimulating hormone (FSH). LH, in particular, directly stimulates the Leydig cells in the testes to enhance the production of testosterone (Plant and Marshall 2001). Disruptions in the hypothalamic-pituitary axis can impact spermatogenesis and fertility, with effects that may be either transient or severe (Singh et al. 2019).

Several case reports have documented conditions such as testicular hypoplasia; testicular degeneration (TD), intersex conditions, epididymitis, and azoospermia in dogs (Clough et al. 1970; Brown et al. 1976; Kelly et al. 1976; Ellington et al. 1993; Metcalfe et al. 1999). Additionally, clinical and epidemiological surveys have addressed the occurrence of testicular tumors and azoospermia in dogs (Hayes and Pendergrass 1976; Nieto et al. 1989; Oguejiofor et al. 2020). Testicular hypoplasia, although a relatively common congenital condition in other domestic species (Blanchard et al. 1990), is rarely reported in dogs. Pathologies of the reproductive organs in dogs, including cryptorchidism and testicular tumors, exhibit epidemiological patterns comparable to those observed in humans (Pendergrass and Hayes 1975; Hayes et al. 1985).

Stray dogs, in particular, are continuously exposed to a range of adverse environmental factors that may accelerate the onset of degenerative testicular changes at a younger age (Ortega-Pacheco et al. 2006). The incidences of TD ranged between 18.3% and 33.3% in the young and old stray dogs, respectively (Ortega‐Pacheco et al. 2006), making it one of the most prevalent reasons for acquired infertility and poor semen quality (Domingos and Salomão 2011; Fontbonne 2011). Moreover, Ortega‐Pacheco et al. (2006) demonstrated that body condition score (BCS) and age were identified as significant risk factors for the development of testicular degeneration, while dog size had no notable influence and did not appear to affect the prevalence of cryptorchidism or testicular hypoplasia. However, older dogs exhibited a higher frequency of testicular tumors. Seminal analysis identifies the main problems, which are connected to poor semen quality, much like in humans. As per the findings of Rijsselaere et al. (2007) and Domosławska et al. (2013), infertile dogs exhibit significantly lower sperm concentration (40.15 ± 36.56 × 10^6^/mL), a lower percentage of spermatozoa with normal morphology (66.4 ± 19.47%), and reduced motility (62.6 ± 30.91%) across most assessed parameters compared to fertile dogs. Testes exhibiting partial or complete degeneration were found to be either azoospermic or oligozoospermic, with the extent of sperm production impairment correlating to the degree of damage to the testicular parenchyma (Ortega‐Pacheco et al. 2006).

The remarkable clinical condition known as oligozoospermia is caused by the testes producing fewer spermatozoa over the course of reproductive life. Oligozoospermia is considered to be one of the most prevalent clinical causes of male infertility in humans, despite the fact that its pathophysiology is still unclear (Haslett et al. 2002; Pramanik 2007). Currently, there isn’t a single drug that works well to treat the condition. In the last ten years, a lot of research has been done on the use of natural sources to treat oligozoospermia (Mukram et al. 2013). Recent studies showed that 15–20% of male dogs kept for breeding had problems related to subfertility (Domosławska et al. 2019).

Semen improvement in male stud dog is a field of permanent interest (Schäfer-Somi 2015). Investigations of different oral supplements have been made with this regard. Most often, supplementation of vitamin E alone (Hatamoto et al. 2006; Da Rocha et al. 2009) or in combination with vitamin C (Lopes-Santiago et al. 2012) was used for significant improvement of ejaculate volume, progressive motility and decreasing of total sperm pathology. The efficacy of a commercially available nutraceutical diet was also tested in male dogs and it was found out that motility percentage, semen volume, concentration and total number of sperms per ejaculation were significantly increased (Ciribé et al. 2018). Speman^®^ is a formulation of plant origin developed by The Himalaya Drug Company (Makali, Bangalore) with no side effects, which has been tested in humans with oligozoospermia, asthenozoospermia, enlarged prostate and azoospermia (Kadhem and Al-Ani 2013), and also in oligozoospermic dogs (Antonov 2024). A combination of extracts from Withania somnifera, Asteracantha longifolia, Lactuca scariola, Mucuna pruriens, Parmelia parlata, Argyreia speciosa, Tribulus terrestris, Leptadenia reticulate, and Suvarnavang make up Speman^®^ (Mukram et al. 2013).

Platelet-rich plasma (PRP) has been extensively utilized in the treatment of various tissue injuries as a source of growth factors. PRP may influence tissue healing by stimulating the growth and maturation of tissue progenitor cells. A volume of plasma with a platelet count above baseline is referred to as PRP (Zhu et al. 2013). Given that the typical platelet count ranges from 150 to 450 × 10^9^/L (Levi et al. 2002). They have a large number of proteins, cytokines kept in cytoplasmic granules, and many growth factors (GFs) (Pietrzak and Eppley 2005). Some proteins, such as GFs, peptide hormones, and chemo attractants for neutrophils, macrophages, and stem cells, as well as several hundred other proteins, including fibrinogen and fibrin, were studied and their physiological functions investigated. Furthermore, several proteins possess antimicrobial and fungicidal properties. Adenosine triphosphate (ATP), adenosine diphosphate (ADP), superoxide dismutase enzyme (SOD), calcium ions, histamine, serotonin, and dopamine are found in the dense granules of platelets and are crucial for maintaining tissue homeostasis. The platelet-derived growth factor (PDGF), vascular endothelial growth factor (VEGF), the epidermal growth factor (EGF), the insulin-like growth factor (IGF-I), the transforming growth factor b-I (TGFb-I), the hepatocyte growth factor (HGF), and the fibroblast growth factor (FGF) are among the various growth factors that are stored and released by platelets (Pietrzak and Eppley 2005).

Under normal physiological conditions, the many components present in platelet granules operate in concert with nearby cells to facilitate the healing of wounds (Lacci and Dardik 2010). As a result of an external or native stimulus, platelet granules go through exocytosis. Native collagen, which is present in the extracellular matrix of almost all human body tissues, is an example of an activating agent (Di Matteo et al. 2015). Following the initial release of GFs during activation, GF levels are maintained three to five times higher than initial values by continuous exocytosis (Zhu et al. 2013).

Platelet-rich plasma has demonstrated several therapeutic benefits across various medical and veterinary applications. It has been shown to aid in the treatment of large cutaneous defects and accelerate delayed wound healing (Kim et al. 2009). In cases of full-thickness burn wounds, the use of autologous PRP has been linked to faster healing, shorter recovery times, and a reduction in secondary complications associated with the burn injury (Lee et al. 2018). In veterinary orthopedics, intra-articular PRP injections have been found to improve limb function and reduce pain in dogs with osteoarthritis, specifically in a model involving the anterior cruciate ligament (Cook et al. 2016). Moreover, PRP has proven effective in managing chronic lameness in dogs suffering from degenerative joint disease of the stifle, a condition resulting from cranial cruciate ligament rupture (Vilar et al. 2018). Additionally, PRP plays a role in enhancing the maturation of grafts during anterior cruciate ligament reconstruction by promoting processes like revascularization and reinnervation (Xie et al. 2013). Finally, PRP has been utilized as an adjunctive treatment in revascularization procedures for managing immature teeth in dogs, improving the success rates of these procedures (Stambolsky et al. 2016). Because PRP contains supra-physiological biological growth factors, it has been successfully employed in reproductive biology and tissue regeneration in both human and veterinary medicine (Nazari et al. 2016; Cremonesi et al. 2020; Dawod et al. 2021). Numerous of these factors were only examined in relation to spermatozoa, demonstrating a markedly positive effect on both their quality and function. Sperm motility was shown to be enhanced by TGF â, FGF, VEGF, Zn, and SOD, but normal sperm parameters were linked to IGF- 1 and PDGF (Borrione et al. 2010; Ding et al. 2011; Bader et al. 2019).

Platelet-rich plasma, which demonstrated an improvement in vitality and motility as well as a significant decrease in vacuolization, DNA fragmentation, and ROS levels, should be considered to be a promising therapy due to its high efficacy, safety (Bader et al. 2019) and its role in solving some male fertility problems such as: azoospermia (Al-Nasser et al. 2018) and oligozoospermia (Fazli et al. 2024). Because of its regenerative properties, PRP is becoming more and more popular in reproductive medicine. Studies on PRP’s effects on male infertility are still in their early stages. In testicular torsion, PRP has a positive impact on spermatogenesis and the generation of reproductive hormones (Lubkowska et al. 2012; Kutluhan et al. 2021). In a recent study, the administration of PRP enhanced the efficacy of busulfan treatment, mitigating structural and functional impairments of the testes. This improvement was associated with an increase in the number of spermatogenic stem cells, as well as enhancements in sperm count, motility, tail length, and testosterone levels (Dehghani et al. 2018). Furthermore, subcutaneous injection of PRP in the scrotal area was found to rejuvenate testicular microstructure, restore the integrity of the spermatogenic epithelium, and normalize both the quantity and function of Sertoli cells in mice with doxorubicin-induced testicular hypofunction (Valerıya et al. 2014).

Another experiment demonstrated that PRP administration effectively corrected alterations in the activities of SOD, caspase- 3, and myeloperoxidase, as well as levels of malondialdehyde and serum testosterone following testicular ischemia/reperfusion injury (Sekerci et al. 2017). Research by Saba et al. (2023) demonstrated that PRP treatment leads to the renewal of testicular microstructure and facilitates tubule regeneration, mediated by the cytokines and growth factors present in PRP. Additionally, both human and animal studies have indicated that PRP contributes to the morpho-functional restoration of the testes (Al-Nasser et al. 2018).

Because of its supra-physiological biological properties, autologous PRP has been effectively utilized in reproductive biology and tissue regeneration in both human and veterinary medicine (Aghajanzadeh et al. 2020; Farghali et al. 2022). PRP has recently been added to semen diluent for the preservation of human and goat semen in frozen and refrigerated environments, with promising outcomes and an enhancement in the quality of post-thaw semen (El-Sherbiny et al. 2022a). In contrast, a little is known about how autologous PRP affects the quality of semen and testicular blood flow in dogs. Therefore, the aim of this study is to investigate the impact of autologous PRP used for treatment of oligozoospermia in dogs on the quality of semen and testicular blood flow.

## Materials and methods

### Experimental location and ethical approval

This study was conducted at the dog stud of the Department of Theriogenology, Faculty of Veterinary Medicine, Cairo University (latitude 30°01’ N; longitude 31°21’ E), from October, 2023, to November, 2023. The study received ethical approval from the institutional animal care committee of the Faculty of Veterinary Medicine, Cairo University (Approval No.: Vet CU 18042024892).

### Animals and management

Ten stray male dogs (30 ± 0.5 kg BW, age: 2 ± 0.5 years) of unknown breeding history were included in this study. Before starting the experiment, a standard physical examination, along with a comprehensive assessment of the reproductive tract, was performed. All animals were tested for Brucella canis using the antigen rapid canine Brucella AB test (FASTest^®^ BRUCELLA Canis) as described by manufacturers and the result was negative for Brucella canis in all animals in the study. All animals appeared healthy, with no obvious physical or reproductive abnormalities. This was followed by ultrasonography scanning of the male genital organs—including the testes, epididymis, and prostate—as described elsewhere (Alonge et al. 2018a, b). All organs showed normal texture, size, and echogenicity, as outlined by Bracco et al. (2023). Prior to PRP administration, all males underwent monthly semen collection and evaluation as all males suffered from oligozoospermia confirmed by semen collection and semen picture assessment. The result of their semen analysis was that the concentration (x10^6^/ml) was 52.0 ± 1.30, and the motility% was 46.0 ± 1.87. In comparison, the control dogs had a sperm concentration (10^6^/ml) of 53.2 ± 1.15 and motility% of 49.0 ± 1.87. Based on these results, the study dogs were classified as oligozoospermic, following the criteria outlined in a recent study by Antonov (2024), who defined oligozoospermic dogs as having a concentration (10^6^/ml) of 55.43 ± 13.12 and motility% of 80.04 ± 4.51.

Dogs were randomly divided into two groups. Group I is the control group (*n* = 5), without any treatment, and Group II is the autologous platelet-rich plasma group (PRP) (*n* = 5). Every animal involved in the study was housed indoors, given regular exercise, a commercial diet consisting of dehydrated poultry protein, maize, maize flour, animal fats, wheat, rice, maize gluten, hydrolysed animal proteins, beet pulp, fish oil, soya oil, minerals, yeasts and its components, hydrolysed crustaceans (source of glucosamine), hydrolysed cartilage (source of chondroitin), and allowed unlimited access to water.

### Experimental design

Males were subjected to routine examination (every 2 weeks, 2 times per month) at weeks 0, 2, 4, 6, and 8 to determine the effect of a single intra-testicular injection of the autologous PRP on testicular hemodynamics and semen analysis compared to other non-injectable animals serving as controls. Dogs were subjected to blood collection, and ultrasonography examination at weeks 0, 2, 4, 6, and 8. Semen collection was done at day 0, 28, and 60. The assessment was performed on day 0 before injection, and the examination was extended to 8 weeks.

### PRP preparation and injection

Their autologous blood samples were used to create the PRP in accordance with established standard procedures (Dhurat and Sukesh 2014). In short, to avoid clotting, 5 ml of whole blood was drawn into tubes containing EDTA, an anticoagulant, and gently mixed. After that, the blood was centrifuged for 5 min at 3000 rpm, causing it to separate into distinct layers. The layers that accumulated were red blood cells at the bottom, white blood cells and platelets in the middle layer, called the buffy coat, and plasma at the top. The top layer (plasma) was carefully transferred to a fresh, clean tube using a sterile pipette or syringe. After that, this plasma was centrifuged for 15 min at 3500 rpm. After removing the upper two-thirds, which were made of platelet-poor plasma (PPP), the lowest one-third, which included PRP, remained. 23 µl of 10% calcium chloride were added, and the PRP was then incubated for 15 min at 37 °C. To obtain activated PRP, the tube was centrifuged for 10 min at 4000 rpm following incubation.

**Fig. 1:**
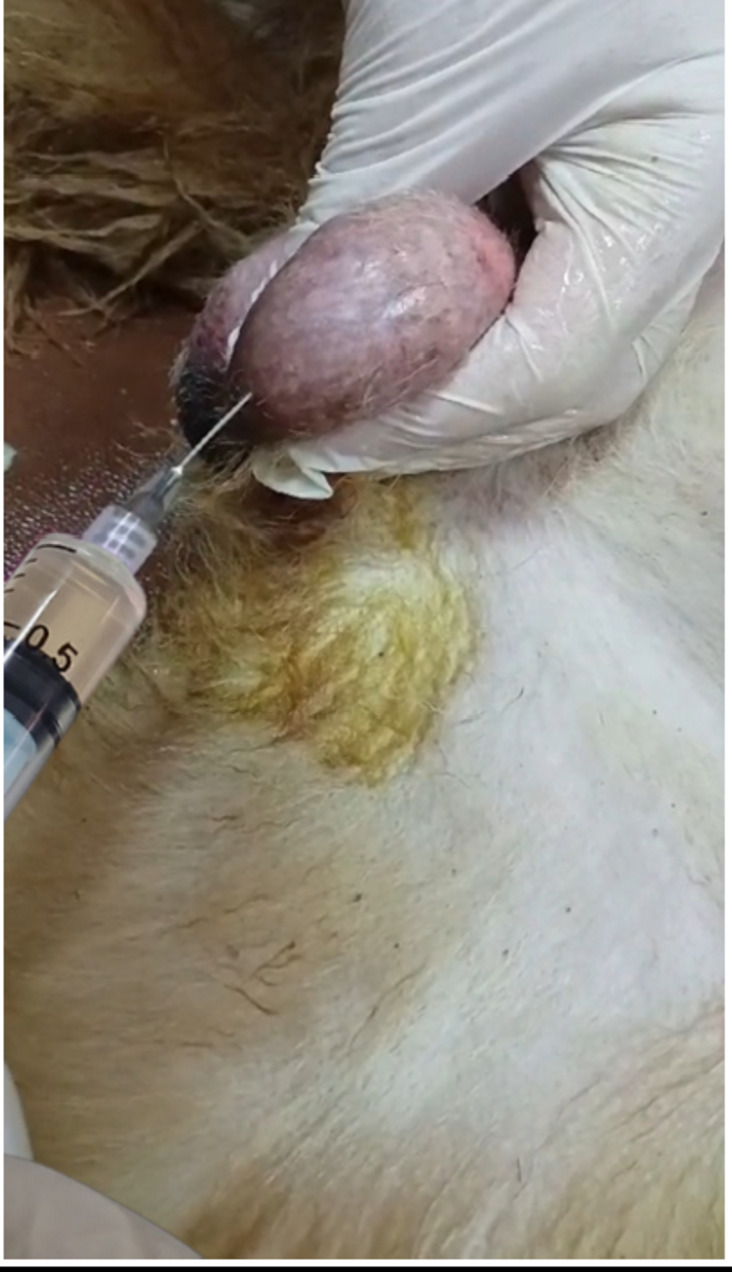
Injection of PRP into the testicle

The dogs were given intramuscular injections of xylazine (1.0 mg/kg) and ketamine (1.0 mg/kg) in accordance with Silva et al. (2020). The testicles were clipped and sterilized with 70% ethyl alcohol prior to injection. Each intratesticular injection was carried out using a sterile 21-gauge needle. The needle was inserted from the codoventral side of the testis, about 1 cm from the epididymal tail, and directed towards the dorsocranial side as shown in Fig. 1. The solution was gradually injected along the entire length of the testis by withdrawing the needle from the proximal (closer to the body) to the distal (further from the body) end, ensuring even distribution of the solution (Jana and Samanta 2007). Every testicle received a single injection of 0.5 ml of PRP (Al-Nasser et al. 2018).

### Ultrasonography evaluation

**Fig. 2:**
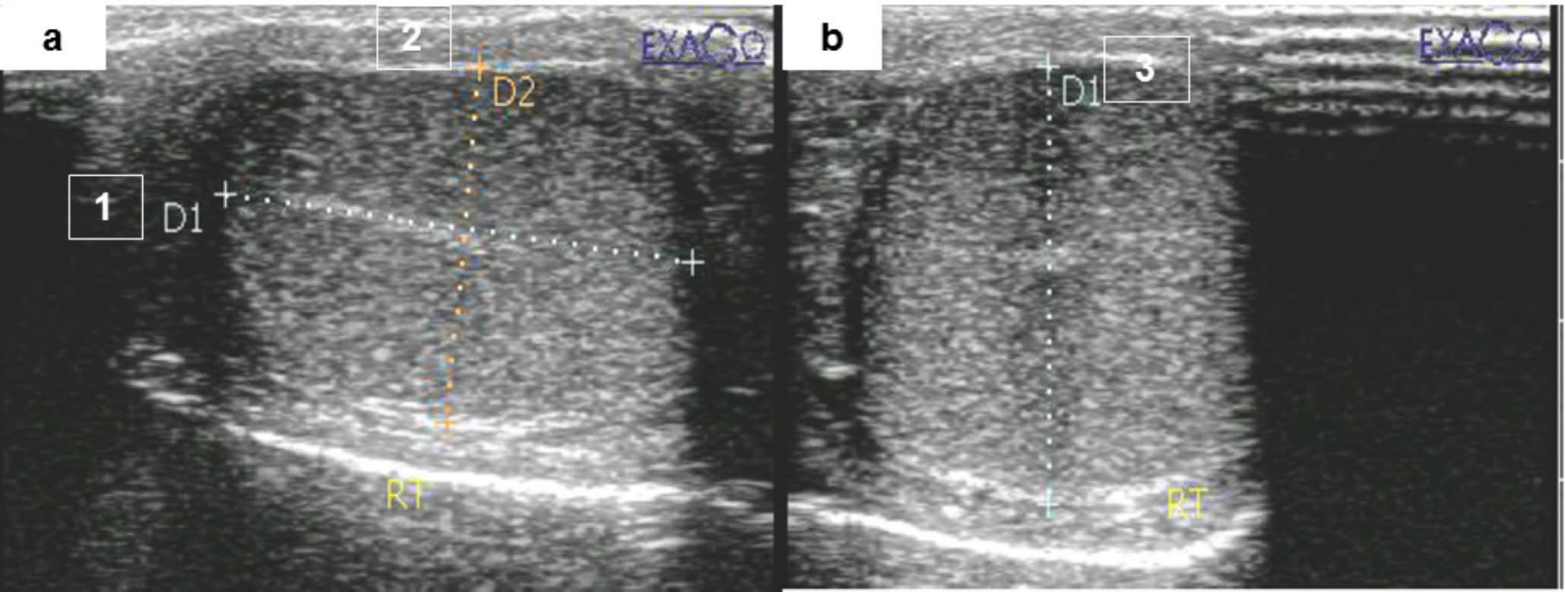
Ultrasonography revealed the testicular dimensions to estimate the testicular volume using the ellipsoid shape formula. Length (1) and height (2) are measured in the longitudinal scan (**a**), while the testicular width (3) is measured in the transverse scan (**b**)

Each dog’s right and left testicles were examined with an EXAGO Doppler ultrasound device (EXAGO, Echo Control Medical, Angoulême, France) equipped with a linear array transducer capable of frequencies ranging from 5 to 9 MHz. The dogs were positioned dorsal recumbent, and after applying acoustic gel to the skin, the transducer was first positioned on the lateral surface of the testis. Using longitudinal and transverse B-mode imaging, testicular length and width were measured, with the mediastinum acting as a reference point. Assessment was done using the ellipsoid formula, V = length x width x height x 0.5236, to determine the testicular volume (de Souza et al. 2015) as shown in Fig. 2.

### Measurement of the testicular artery doppler parameters

The three best continuous waves with complete systolic and diastolic endpoints were used to determine all Doppler velocimetry measurements, including peak systolic velocity (PSV; cm/sec), end-diastolic velocity (EDV; cm/sec), and both Doppler indices expressed by the resistance index (RI) and pulsatility index (PI), which are the most commonly used (Maher et al. 2020; Abdelnaby et al. 2022). For measuring testicular artery flow, color Doppler ultrasound was employed with the transducer initially placed at the neck of the scrotum to locate the tortuous distal (looping) segment of the supratesticular artery, just cranial to the cranial pole of the testis.

The color gain was adjusted to minimize excess color noise, and the pulse-wave Doppler gate was positioned within the vessel lumen. Three cardiac cycles were used to obtain mean values for PSV and EDV, which was then utilized by the machine’s software to calculate the RI and PI. The operator and machine settings (depth: 4.5–5.5 cm; pulse repetition frequency: 2.5 kHz; wall filter: 5 cm/s; sample gate: 2.0 mm) were kept consistent across all examinations. The angle between the Doppler beam and the vessel’s long axis was maintained at less than 60°, with angle corrections applied as necessary. In most instances, an angle of 0° was used (de Souza et al. 2015).

### Semen collection and evaluation

Manual manipulation was utilized to collect semen in accordance with Linde-Forsberg (1991). The ejaculates were collected into a cup that has been preheated to 36–38 °C. Two collections were made after 28, 60 days following therapy, and one collection occurred prior to the intra-testicular injection of PRP. A hemocytometer and a light microscope were used to measure the concentration of sperm cells (Mickelsen et al. 1993). The percentage of motility of the obtained sperm was subjectively assessed to the nearest 5% immediately after collection using a phase-contrast microscope with a heated stage (37 °C) and 200× magnification (El-Sherbiny et al. 2022a). Every sample was examined by two observers, and variations within samples were never more than 5%. The percentage of living and dead spermatozoa was calculated using dried smears stained with eosin/nigrosin (Campbell et al. 1956; Johnston et al. 2001b). A total of 200 spermatozoa were checked in randomly selected fields under oil immersion lens (100X) of phase contrast microscope to evaluate sperm abnormalities.

### Blood sampling and hormonal assaying

Two milliliters of blood were collected at 9 AM for a fortnightly interval from the cephalic vein one hour after the intravenous injection of Receptal^®^ (GnRH analogue, Intervet Deutschland GmbH, Germany) at a dosage of 0.4 µg/kg B.W (Spruijt et al. 2023). Centrifugation at 3,000 g for 5 min at room temperature obtained the serum (Domosławska and Zdunczyk 2020). Prior to using serum samples for hormonal analysis, they were kept in storage at − 20 °C. Serum testosterone (T) was measured by commercial ELISA kits (DiaSino Laboratories Co., Ltd., Zhengzhou, China) as described by manufacturers. The intra- and inter-assay coefficients of variation, respectively, were 3.3 and 4.8%. The assay sensitivity was 0.05 ng/ml. Using a spectrophotometer (Prietest Touch, Robonik, India) at 540 nm, nitric oxide was measured in serum samples using commercial kits (nitric oxide kit, Bio-diagnostic, Dokki, Egypt) following the manufacturer’s instruction. The NO intra-assay coefficient variation was 5.3%, with an assay sensitivity of 0.225µmol/l in nitrite form.

### Statistical analyses

This study was conducted with a sample size of 5 dogs per group, which was deemed adequate to detect biological differences between groups and ensure statistical significance, while remaining consistent with ethical standards and the resources available at the time of the experiment. The sample size was determined through a power analysis test (Chow et al. 2008; Cesana and Antonelli 2016). The data were initially examined for normality using the Kolmogorov-Smirnov test, and the results indicated that the data were normally distributed. To differentiate between the means at the various time points under study, the repeated measures ANOVA test was employed, followed by the Bonferroni post hoc test. Comparison between the two groups is done using a t-test (student’s unpaired t-test). All of the statistical analyses that were examined were conducted using GraphPad Prism5. *P* < 0.05 was considered statistically significant.

## Results

### B-mode ultrasonic findings of volume of dog testes

The information displayed in Table 1 illustrates the variations in the volume of dog testes measured using B-mode ultrasonography. It was observed that no significant difference in testicular volume was detected between the two groups at the start of the experiment and up until the 4^th^ week. However, a time-dependent increase in testicular volume was noted, with the maximum volume observed in Group II after the 6^th^ and 8^th^ weeks of the study, compared to other time points.

**Table 1:** The effect of a single intra-testicular injection of the autologous PRP on the volume of testes (cm^3^) (Mean ± SEM)

		Duration of the study				
Groups	Traits	Day 0	2^nd^ week	4^th^ week	6^th^ week	8^th^ week
	Volume of right testes	6.13 ± 0.22^A,a^	6.14 ± 0.22^A,a^	6.10 ± 0.22^A,a^	6.13 ± 0.23^A,a^	6.13 ± 0.23^A,a^
Control (n=5)						
	Volume of left testes	6.68 ± 0.11^A,a^	6.68 ± 0.26^A,a^	6.74 ± 0.15^A,a^	6.81 ± 0.11^A,a^	6.83 ± 0.11^A,a^
	Volume of right testes	6.71 ± 0.24^A,a^	6.69 ± 0.22^A,a^	6.96 ± 0.32^A,a^	8.08 ± 0.35^B,b^	8.51 ± 0.39^B,b^
PRP (n=5)						
	Volume of left testes	7.96 ± 0.33^A,a^	8.02 ± 0.46^A,a^	8.05 ± 0.34^A,a^	8.76 ± 0.54^B,a^	9.23 ± 0.64^B,b^

Mean with different superscripts (A, B) within the same column, (a, b) within rows were significantly different at *P* < 0.001

### Testicular hemodynamics

**Fig. 3:**
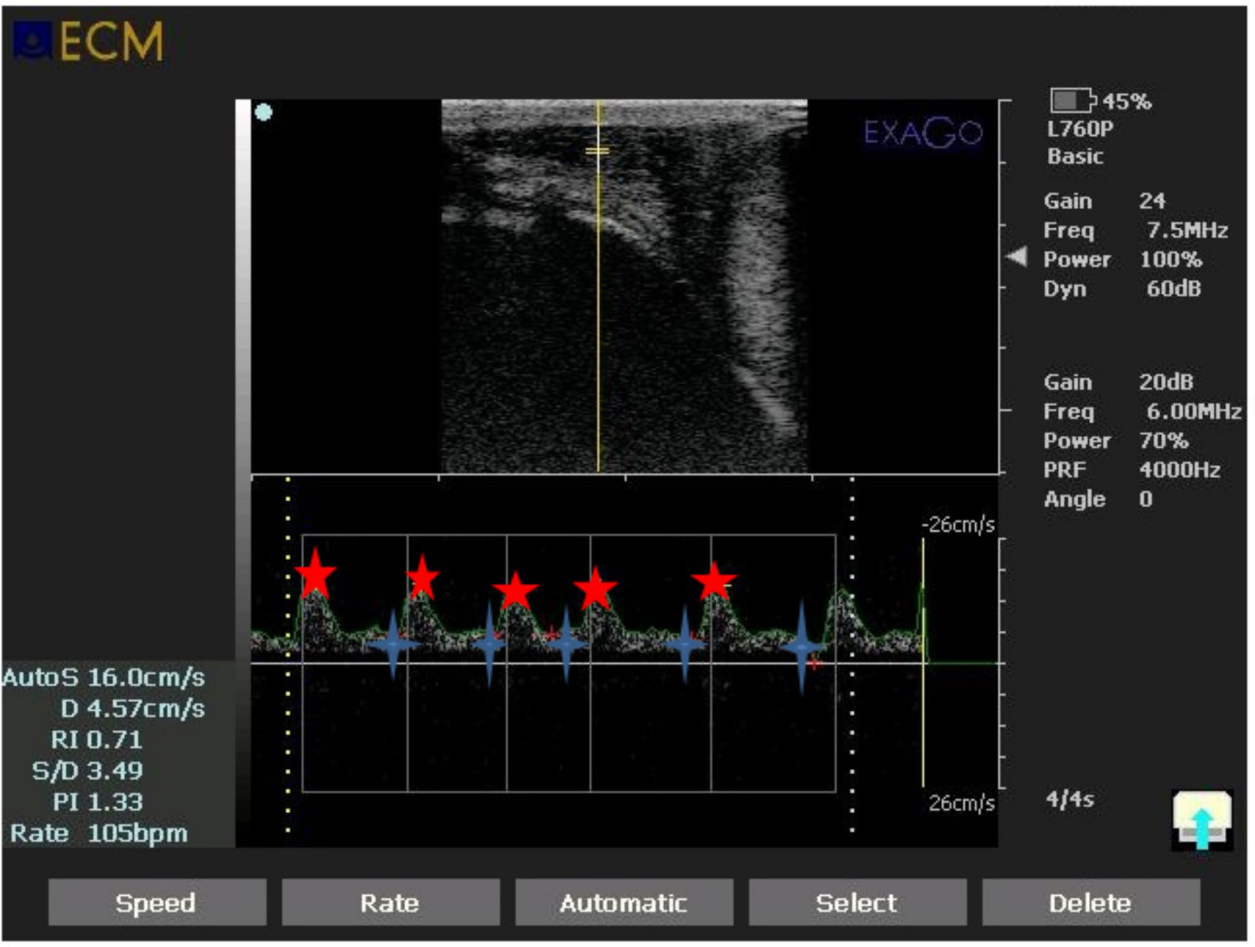
Pulsed-wave Doppler ultrasonography images of the testicular artery at 0 day before injection with the automatic calculation of both Doppler indices (RI and PI). Red star showed the Peak Systolic Velocity (PSV; cm/sec), while the blue asterisk showed the End Diastolic Velocity (EDV; cm/sec). RI = resistive index, PI = pulsatility index

**Fig. 4:**
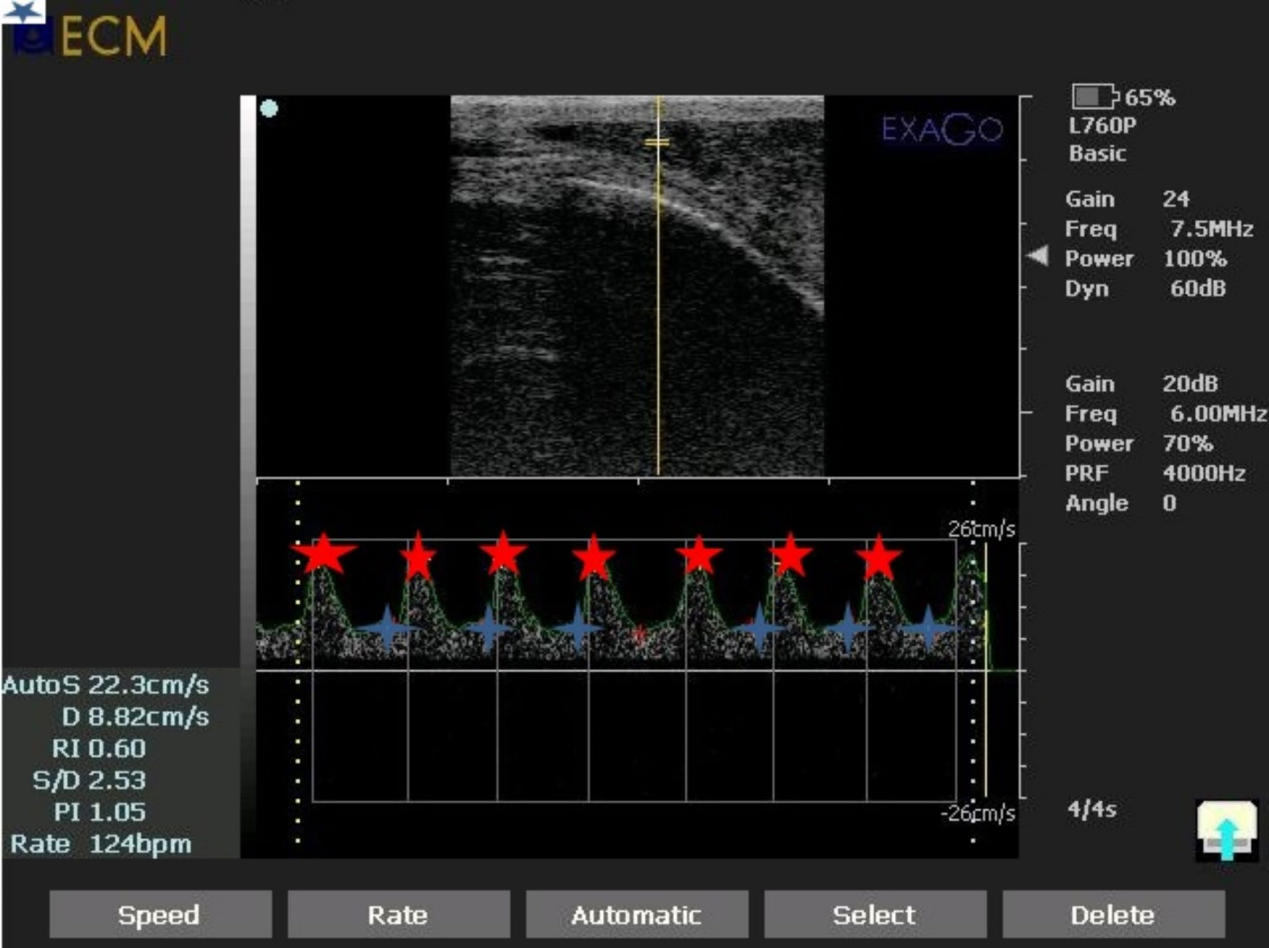
Pulsed-wave Doppler ultrasonography images of the testicular artery 8 weeks post-injection with the automatic calculation of both Doppler indices (RI and PI). Red star showed Peak Systolic Velocity (PSV; cm/sec), while the blue asterisk showed End Diastolic Velocity (EDV; cm/sec). RI = resistive index, PI = pulsatility index

The results presented in Table 2 show changes in testicular blood flow. At the start of the experiment, no significant difference in testicular blood flow was observed between the two groups, as illustrated in Fig. 3. However, a single intra-testicular injection of PRP resulted in a highly significant (*P* < 0.01) increase in EDV and PSV compared to the control untreated group, starting at 2 weeks and continuing through the end of the study at 8 weeks. Furthermore, dogs in group II exhibited significantly higher EDV and PSV values (*P* < 0.001) from the 6^th^ to 8^th^ week post-injection, compared to baseline values recorded on day 0, before treatment. Group II showed significantly lower RI and PI values (*P* < 0.001) from the 2^nd^ to 8^th^ weeks following injection, compared to Group I. A highly significant (*P* < 0.001) decrease in both RI and PI was also observed from the 2nd to 8^th^ weeks, relative to the baseline values obtained on day 0 (Fig. 4).

**Table 2:** The effect of a single intra-testicular injection of the autologous PRP on testicular hemodynamic (Mean ± SEM)

		Duration of the study				
Groups	Measures	Day 0	2^nd^ week	4^th^ week	6^th^ week	8^th^ week
	PSV(cm/sec)	19.96 ± 0.33^A, a^	19.80 ± 0.35^A, a^	20.85 ± 0.29^A, a^	20.94 ± 0.40^A, a^	21.16 ± 0.18^A, a^
	EDV(cm/sec)	4.79 ± 0.32^A, a^	4.34 ± 0.14^A, a^	4.41 ± 0.18^A, a^	4.99 ± 0.28^A, a^	5.15 ± 0.21^A, a^
Control (n=5)	RI	0.74 ± 0.00^A, a^	0.76 ± 0.01^A, a^	0.77 ± 0.00^A, a^	0.77 ± 0.00^A, a^	0.76 ± 0.00^A, a^
	PI	1.64 ± 0.04^A, a^	1.58 ± 0.08^A, a^	1.61 ± 0.05^A, a^	1.60 ± 0.06^A, a^	1.64 ± 0.04^A, a^
	PSV(cm/sec)	21.8 ± 0.65^A, a^	22.6 ± 0.71^B, a^	23.8 ± 1.20^B, a,b^	24.0 ± 0.49^B, a,b^	24.8 ± 0.44^B, b^
PRP (n=5)	EDV(cm/sec)	5.77 ± 0.31^A, a^	6.35 ± 0.47^B, a^	6.40 ± 0.56^B, a^	8.25 ± 0.45^B, b^	8.67 ± 0.62^B, b^
	RI	0.74 ± 0.00^A, a^	0.70 ± 0.00^B, a^	0.63 ± 0.00^B, c^	0.61 ± 0.00^B, c,d^	0.59 ± 0.01^B, d^
	PI	1.51 ± 0.06^A, a^	1.37 ± 0.00^B, a^	1.16 ± 0.02^B, b^	1.09 ± 0.01^B, b^	1.00 ± 0.04^B, b^

Mean with different superscripts (A, B) within the same column, (a, b, c, d) within rows were significantly different at *P* < 0.001

### Semen analysis

The effects of PRP on dog sperm parameters are summarized in Table 3. Statistical analysis revealed a significant increase in sperm cell concentration, motility percentage, live spermatozoa, and normal spermatozoa (*P* < 0.001) starting from day 28, with the highest values observed at day 60 in Group II compared to the untreated control group. Additionally, the percentage of sperm cell abnormalities decreased significantly (*P* < 0.001) at both day 28 and day 60 following PRP treatment in dogs with oligozoospermia, when compared to Group I. A time-dependent increase in sperm cell concentration, motility, and the percentages of live and normal spermatozoa was noted, with peak values occurring at 28 and 60 days post-treatment, compared to baseline values recorded on day 0.

**Table 3:** The effect of a single intra-testicular injection of the autologous PRP on sperm cell concentration (x10^6^/ml), motility%, live/dead ratio, and abnormalities % in dogs (Mean ± SEM)

		Duration of the study		
Groups	Measures	Day 0	Day 28	Day 60
	Sperm cell concentration (x10^6^/ml)	53.2 ± 1.15^A, a^	55.4 ± 1.69^A, a^	56.4 ± 0.07^A, a^
Control (n=5)	Motility %	49.0 ± 1.87^A, a^	48.0 ± 1.22^A, a^	48.0 ± 1.23^A, a^
	L/D ratio	89.0 ± 1.18^A, a^	89.4 ± 0.87^A, a^	87.0 ± 0.70^A, a^
	Abnormalities%	12.0 ± 0.70^A, a^	11.4 ± 0.40^A, a^	11.6 ± 0.50^A, a^
	Sperm cell concentration (x10^6^/ml)	52.0 ± 1.30^A, a^	236.2 ± 0.86^B, b^	296.2 ± 1.68^B, c^
PRP (n=5)	Motility %	46.0 ± 1.87^A, a^	63.4 ± 0.92^B, b^	72.4 ± 1.02^B, c^
	L/D ratio	91.2 ± 0.58^A, a^	92.8 ± 0.86^B, a,b^	95.4 ± 0.50^B, b^
	Abnormalities%	11.6 ± 0.50^A, a^	7.6 ± 0.50^B, b^	7.0 ± 0.70^B, b^

Mean with different superscripts (A, B) within the same column, (a, b, c) within rows were significantly different at *P* < 0.001

### Serum testosterone and NO levels

At the start of the experiment, there were no significant differences between the two groups in serum testosterone concentration or NO levels. The circulating serum testosterone and NO levels ranged from 2.7 ± 0.05 to 2.8 ± 0.0 ng/ml and 30.58 ± 0.18 to 30.86 ± 0.21 µmol/ml, respectively. However, two weeks after the initiation of the single intra-testicular autologous PRP treatment, serum testosterone concentrations and NO levels were significantly higher (*P* < 0.001) in Group II compared to both baseline values (day 0) and Group I. These elevated levels persisted throughout the 8-week study period, as shown in Tables 4 and 5.

**Table 4:** The effect of a single intra-testicular injection of the autologous PRP on the serum level of testosterone (ng/ml) in dogs (Mean ± SEM)

	Duration of the study				
Groups	Day 0	2^nd^ week	4 ^h^ week	6^th^ week	8^th^ week
Control (n=5)	2.72 ± 0.05^A, a^	2.28 ± 0.07^A, a^	2.29 ± 0.07^A, a^	2.29 ± 0.07^A, a^	2.31 ± 0.06^A, a^
PRP (n=5)	2.87 ± 0.00^A, a^	3.72 ± 0.00 ^B, b^	4.32 ± 0.00 ^B, c^	5.05 ± 0.01^B, d^	5.76 ± 0.00^B, e^

Mean with different superscripts (A, B) within the same column, (a, b, c, d, e) within rows were significantly different at *P* < 0.001

## Discussion

Practitioners are looking for specific treatments to deal with subfertility, which is a major problem in canine reproduction. Subfertility is a serious problem that is frequently associated with low sperm concentration and function, both of which are important breeding limitations for dogs. Although several protocols have been investigated to improve these parameters, little is known about how autologous PRP affects the quality of semen and testicular blood flow in dogs. Because of its regenerative properties, PRP is becoming more and more popular in reproductive medicine. Studies on PRP’s effects on male infertility are still in their early stages, despite the fact that a great deal of research has been done on its benefits for female reproductive health, including helping women with recurrent implantation failure (Aghajanzadeh et al. 2020) and returning normal endometrial function in mares after endometritis (Farghali et al. 2022).

**Table 5:** The effect of a single intra-testicular injection of the autologous PRP on the serum level of nitric oxide (µmol/ml) in dogs (Mean ± SEM)

	Duration of study				
Groups	Day 0	2^nd^ week	4^th^ week	6^th^ week	8^th^ week
Control (n=5)	30.58 ± 0.18^A, a^	31.04 ± 0.15^A, a^	30.93 ± 0.02^A, a^	31.54 ± 0.2^A, a^	31.55 ± 0.17^A, a^
PRP (n=5)	30.86 ± 0.21^A, a^	33.35 ± 0.17^B, b^	36.45 ± 0.14^B, c^	41.34 ± 0.2^B, d^	67.12 ± 0.06^B, e^

Mean with different superscripts (A, B) within the same column, (a, b, c, d, e) within the same row were significantly different at *P* < 0.001

Platelet-rich plasma is used clinically because of its ability to increase the concentration and production of proteins and growth factors that promote cellular healing processes (Amable et al. 2013). It has been demonstrated that PRP accelerates vascularization, lowers post-surgical morbidity, enhances tissue regeneration, and minimizes the formation of scars (Lacci and Dardik 2010). Additionally, it promotes wound healing by accelerating the maturation and regeneration of epithelial cells (Oneto and Etulain 2021). According to Kevy and Jacobson (2004) and Foster et al. (2009), PRP is also believed to improve tissue regeneration by promoting the recruitment, proliferation, and differentiation of cells. One benefit of using autologous PRP is that it removes the possibility of immunological responses or microbial infections spreading, as well as the risk of cross-contamination (Marx et al. 1998).

The results demonstrated that a single intra-testicular injection of autologous PRP significantly (*P* < 0.001) increased sperm count, motility, and the proportion of live and normal spermatozoa in oligozoospermic dogs. These findings are consistent with previous research in humans, where PRP treatment has been shown to improve sperm concentration and motility while reducing DNA fragmentation in men with oligozoospermia (Fazli et al. 2024). The growth factors found in PRP, including FGF, TGF-β, IGF, PDGF, and VEGF, may be responsible for the observed improvement in sperm parameters. These growth factors can improve sperm motility, production, and DNA integrity because of their regenerative properties (Anbari et al. 2021; Somova et al. 2021; Doostabadi et al. 2022). Studies have demonstrated the significance of brain-derived neurotrophic factor (BDNF) in the male reproductive system, which has a receptor on sperm (Nilsson et al. 2020; Beitia et al. 2023). The phosphatidylinositol 3 kinase (PI3 K) pathway is activated by BDNF and may be essential for improving DNA integrity and sperm motility (Saucedo et al. 2015). Sperm motility and linear velocity have been demonstrated to be positively affected by VEGF in particular (Hajipour et al. 2021). Furthermore, PRP (5%) was shown to significantly (*P* < 0.05) enhance post-thaw buffalo sperm quality, including progressive motility, membrane (structural and functional) and acrosome integrity, and normal sperm percentages (El-Sherbiny et al. 2022a).

Moreover, PRP was found to improve testicular hemodynamics recorded herein, as evidenced by increased blood velocity indices (PSV and EDV) and decreased PI and RI of the testicular arteries, leading to enhanced testicular vascular perfusion. The improvement in testicular hemodynamics and increased NO levels observed in the present study could be attributed to the growth factors present in PRP. VEGF enhances angiogenesis, promotes endothelial cell mitosis, and improves vascular permeability (Steenfos 1994; Rhee et al. 2004). As a polypeptide, VEGF is essential to the angiogenesis process. It has been established that the male reproductive system contains VEGF and its receptors. The VEGF protein has been found in spermatids, seminal plasma, Sertoli, and Leydig cells, among other places (Nafchi et al. 2022). The vasodilatory effect of VEGF, likely resulting from its increased release following PRP injection, could be a primary factor contributing to the observed therapeutic effects (Bir et al. 2011; Karayannopoulou et al. 2014).

Additionally, NO level was significantly higher (*P* < 0.001) in Group II compared to both baseline values (day 0) and Group I. Thus, as previously shown in dogs, NO directly contributes to the initiation of the vasodilation process in the reproductive system (Salama et al. 2022).These changes suggest improved oxygen and nutrition delivery to the testes and decreased blood flow resistance (El-Sherbiny et al. 2022b).

Furthermore, our results showed that testosterone levels in PRP-treated dogs were considerably significantly elevated (*P* < 0.001) than in the control group after 2 weeks of the treatment till the end of the experiment (8 weeks).This result is consistent with some studies that have proven that intra-testicular PRP administration is significantly associated with elevated testosterone levels; improvements in Leydig cell function, and enhanced testicular steroidogenesis (Somova et al. 2021; Kutluhan et al. 2021; Farhan et al. 2022). The elevated testosterone concentration resulted in an improvement in semen quality because testosterone is a crucial regulator of the structural morphology and proper physiology of seminiferous tubules (Jegou and Sharpe 1993). This study was limited to a small-scale experiment due to resource constraints; future research with a larger sample size is needed to validate these findings and provide more robust conclusions.

## Conclusion

In conclusion, this study is the first to investigate the use of platelet-rich plasma (PRP) in treating oligozoospermia in dogs. The findings are important for improving male dog fertility and reproductive performance. The quality of semen, testicular blood flow, as well as serum levels of testosterone and NO in dogs can all be improved with a single intra-testicular injection of PRP, providing a prospective treatment for subfertility in canine reproduction. This study was limited to a small-scale experiment due to resource constraints; future research with a larger sample size is needed to validate these findings and provide more robust conclusions.

## Data Availability

No datasets were generated or analysed during the current study.
